# External validation of a convolutional neural network for the automatic segmentation of intraprostatic tumor lesions on ^68^Ga-PSMA PET images

**DOI:** 10.3389/fmed.2023.1133269

**Published:** 2023-02-23

**Authors:** Samuele Ghezzo, Sofia Mongardi, Carolina Bezzi, Ana Maria Samanes Gajate, Erik Preza, Irene Gotuzzo, Francesco Baldassi, Lorenzo Jonghi-Lavarini, Ilaria Neri, Tommaso Russo, Giorgio Brembilla, Francesco De Cobelli, Paola Scifo, Paola Mapelli, Maria Picchio

**Affiliations:** ^1^Department of Medicine and Surgery, Vita-Salute San Raffaele University, Milan, Italy; ^2^Department of Nuclear Medicine, IRCCS San Raffaele Scientific Institute, Milan, Italy; ^3^School of Medicine and Surgery, University of Milano–Bicocca, Monza, Italy; ^4^Department of Radiology, IRCCS San Raffaele Scientific Institute, Milan, Italy

**Keywords:** PSMA, convolutional neural network, segmentation, prostate cancer, external validation

## Abstract

**Introduction:**

State of the art artificial intelligence (AI) models have the potential to become a “one-stop shop” to improve diagnosis and prognosis in several oncological settings. The external validation of AI models on independent cohorts is essential to evaluate their generalization ability, hence their potential utility in clinical practice. In this study we tested on a large, separate cohort a recently proposed state-of-the-art convolutional neural network for the automatic segmentation of intraprostatic cancer lesions on PSMA PET images.

**Methods:**

Eighty-five biopsy proven prostate cancer patients who underwent ^68^Ga PSMA PET for staging purposes were enrolled in this study. Images were acquired with either fully hybrid PET/MRI (*N* = 46) or PET/CT (*N* = 39); all participants showed at least one intraprostatic pathological finding on PET images that was independently segmented by two Nuclear Medicine physicians. The trained model was available at https://gitlab.com/dejankostyszyn/prostate-gtv-segmentation and data processing has been done in agreement with the reference work.

**Results:**

When compared to the manual contouring, the AI model yielded a median dice score = 0.74, therefore showing a moderately good performance. Results were robust to the modality used to acquire images (PET/CT or PET/MRI) and to the ground truth labels (no significant difference between the model’s performance when compared to reader 1 or reader 2 manual contouring).

**Discussion:**

In conclusion, this AI model could be used to automatically segment intraprostatic cancer lesions for research purposes, as instance to define the volume of interest for radiomics or deep learning analysis. However, more robust performance is needed for the generation of AI-based decision support technologies to be proposed in clinical practice.

## 1. Introduction

Prostate cancer (PCa) is the second most common cancer in men, with 1,414,259 new cases in 2020, accounting for 15.1% of all cancer diagnoses within the male population (1). Although histopathological examination of prostate biopsy cores is required for the diagnosis of PCa, imaging is pivotal to characterize the disease (2). Multiparametric (mp)-MRI has been used for years in clinical practice to guide biopsy and to drive the clinical management of PCa patients (2).

PSMA PET has been recently added to the EAU-ESTRO-SIOG guidelines for staging high-risk PCa (2) in view of its higher sensitivity compared to mp-MRI (3, 4). Therefore, a possible next step will be to use PSMA PET to diagnose clinically significant PCa (5–8) and to perform quantitative analysis that might allow for a better and more objective characterization of the disease (9–11).

Accurate contouring of intraprostatic gross tumor volume (GTV) is mandatory for an accurate assessment of PCa in several clinical settings, including both biopsy guidance and radiomic features extraction. However, this procedure is time consuming and largely affected by the experience of the contouring physicians, often resulting in non-reproducible segmentations (12).

Recently, there has been a surge in the development of artificial intelligence (AI) models in the medical field, with the first tools being already available for use (13, 14). Convolutional neural networks (CNN) have been shown to accurately segment medical images (15–17) and hold the potential to improve intraprostatic tumor delineation (18–21). The use of CNN in this setting could improve GTV definition by reducing the inter-reader variability while saving time by automating this task.

Kostyszyn and colleagues were the first to develop a CNN for the automatic segmentation of intraprostatic cancer lesions on PSMA (using both ^68^Ga- and ^18^F-PSMA) PET images (18). They used 152 patients examined at two centers (Germany and China) to train their model and a cohort composed by 57 patients to test it. However, only 20 patients in the testing cohort were studied at an external institution (center 3, Germany) not used for training, making it difficult to draw conclusions regarding the model’s generalizability.

External validation of AI models on independent cohorts is necessary to assess with certainty their robustness and reproducibility, hence their possible application in clinical practice (22). Therefore, this study aims to evaluate the performance of the CNN for the automatic segmentation of intraprostatic cancer lesions on ^68^Ga-PSMA PET images that was previously presented in (18) and that is publicly available at https://gitlab.com/dejankostyszyn/prostate-gtv-segmentation.

## 2. Materials and methods

### 2.1. Patients

**FIGURE 1:**
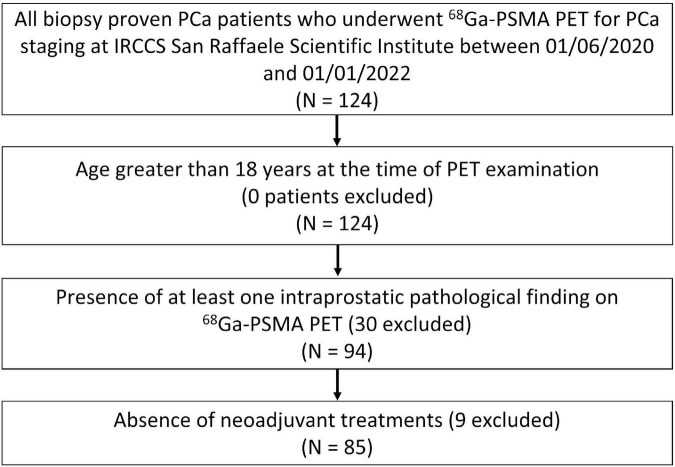
Flowchart illustrating the patients’ selection process.

All patients with biopsy proven PCa who underwent ^68^Ga-PSMA PET at IRCCS San Raffaele Scientific Institute from June 2020 to January 2022 for staging purposes were considered for inclusion. A total of 124 patients was identified. Eligibility criteria were: (1) age greater than 18 years at the time of the PET examination (0 patients excluded), (2) presence of at least one intraprostatic pathological finding at ^68^Ga-PSMA PET (30 patients excluded), (3) absence of neoadjuvant treatments prior to imaging (9 patients excluded). Eighty-five patients met the inclusion criteria and were included for analysis. See Figure 1 for a flowchart showing the patients’ selection process. Prostate specific antigen (PSA) level and the International Society of Urological Pathology (ISUP) grade were collected. This retrospective study was approved by the Institutional Ethics Committee of IRCCS San Raffaele Scientific Institute, and informed consent was waived due to the retrospective nature of the study.

### 2.2. PET imaging

PET scans were acquired using either Signa PET/MRI 3 Tesla system, GE Healthcare, Waukesha, WI, USA (*N* = 46) or PET/CT, Discovery-690, GE Healthcare (*N* = 39).

Fasting condition was requested on the day of ^68^Ga-PSMA PET/MRI and PET/CT scan.

PET scans were acquired from the skull base to mid-thigh (5–6 FOVs, 4 min/FOV), and started approximately 60 min (mean ± SD, 63 ± 6 min) after injection of 111–273 MBq (Mean ± SD, 168 ± 33 MBq) of ^68^Ga-PSMA. PET images, acquired with either PET/MRI or PET/CT scanner, were reconstructed using fully 3D ordered subset expectation-maximization (OSEM) algorithm, time-of-flight (TOF) and point-spread-function (PSF).

^68^Ga PSMA PET image read-out was performed by two Nuclear Medicine physicians on an Advantage Workstation (AW, General Electric Healthcare, Waukesha, WI, USA) and the presence of ^68^GA-PSMA intraprostatic increased uptake was considered positive for malignancy.

### 2.3. Image segmentation

Two Nuclear Medicine physicians manually contoured the GTV on every slice of ^68^GA-PSMA PET images using 3D Slicer (Slicer; version 4.11.2) being aware of all the available patients’ clinical and imaging information. The first reader (Exp 1) delineated the GTV using an inverted gray scale for display, windowed with SUVmin-max: 0–5, as previously described in Kostyszyn at al. (18). To ensure that the segmentation approach used in the reference work was not introducing any bias, a second reader (Exp 2), instead, contoured images independently without any fixed thresholding of voxel values, blind to any instruction on how images were evaluated in the reference work of Kostyszyn et al.

Additionally, two radiologists performed a manual contouring of the prostatic gland on CT and MRI scans by using 3D Slicer (Slicer; version 4.11.2). Since it is not always feasible to discriminate between prostatic tissue and bladder signal in ^68^Ga-PSMA PET images, only contouring within the delinated prostatic gland were used for analyses, as described in Kostyszyn et al.

### 2.4. Resampling

To ensure that the CNN’s performance in this study was not affected by discrepancies in the methods used as compared to the reference work, resampling and preprocessing of the images was performed exactly as described by Kostyszyn et al. (18).

Specifically, all PET images (nearly raw raster data format, nrrd) were resampled to standardize the voxel spacing to 2.0 mm × 2.0 mm × 2.0 mm using SimpleITK (version1.2.4) since the PET images collected with PET/MRI scanner had original voxel size = 3.125 mm × 3.125 mm × 2.780 mm, while the original voxel size of images acquired with PET/CT scanner was 2.734 mm × 2.734 mm × 3.270 mm. Prostate and GTV segmentations were also resampled to a voxel size of 2.0 mm × 2.0 mm × 2.0 mm. PET volumes were resampled using both tri-linear interpolation and B-spline interpolation, whereas Nearest Neighbour interpolation was used to resample segmentation contours. All data were cropped using the manual contouring of the prostate gland as guidance to a size of 64 × 64 × 64 voxels, and then normalized with *x*_*i*_’ $=\frac{x_{i}-\bar{x}}{\sigma }$where *x*_*i*_ is the PET data for patient *i*, and $\bar{x}$ and σ are the arithmetic mean and the standard deviation calculated over the entire cropped PET training dataset.

### 2.5. Convolutional neural network

The model consists of 3 down sampling steps performed by 2 × 2 × 2 max-pooling along the contracting path, and 3 up-sampling steps performed by 2 × 2 × 2 transpose convolutions with padding of 1 and stride of 2 along the expanding paths. Skip connections from the contracting path are concatenated with their corresponding up-sampled feature maps. There are 14 3 × 3 × 3 convolutional layers in total, having stride and padding of 1. Each convolution is followed by batch normalization and ReLU activation function. The last layer in the model performs a 1 × 1 × 1 convolution with no padding, followed by batch normalization and sigmoid activation function. The whole script of the trained CNN can be freely downloaded at https://gitlab.com/dejankostyszyn/prostate-gtv-segmentation.

### 2.6. Statistical analysis

Statistical analyses were performed with R statistical software (23). Dice score coefficient (DSC) was computed to estimate the performance of the trained CNN (GTV-CNN) presented in Kostyszyn et al. (18). Moreover, DSC was also used to quantitatively assess the agreement between the GTVs manually segmented by the different experts (GTV-Exp 1, GTV-Exp 2). As PET volumes in the dataset have been acquired using two different modalities, PET/MRI and PET/CT, Student’s *t*-test was carried out to determine whether the image modality of acquisition possibly affected the model performance. Student’s *t*-test was also employed to determine whether there was a statistically significant difference in CNN performance across the different GTV-Exp segmentations and to study whether the volume predicted by the CNN was different in size as compared to those manually delineated by experts. Ground truth PCa lesion volumes (GTV-Exp) were correlated with DSC scores using Pearson correlation. Finally, to investigate the impact of different interpolation algorithms, analyses were first conducted on PET images resampled using tri-linear interpolation and then on PET volumes resampled with B-spline interpolation. The obtained DSC were compared by means of Student’s *t*-test. *P* values lower than 0.05 were considered statistically significant.

## 3. Results

### 3.1. Patients

**FIGURE 2:**
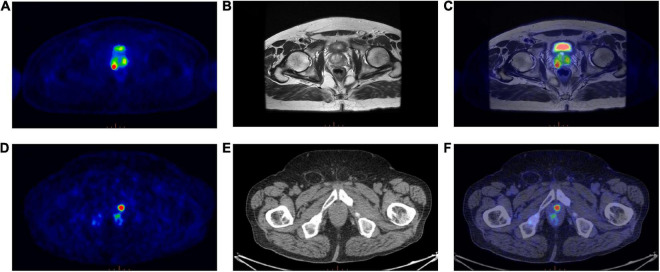
(Top panel) Exemplar image of a ^68^Ga-PSMA PET/MRI scan; **(A)** Transaxial ^68^Ga-PSMA PET, **(B)** Axial T2-weighted MRI sequence, **(C)** ^68^Ga-PSMA PET/MRI. (Bottom panel) Exemplar image of a ^68^Ga-PSMA PET/CT scan; **(D)** Transaxial ^68^Ga-PSMA PET, **(E)** Axial CT image, **(F)** ^68^Ga-PSMA PET/CT.

Eighty-five patients with biopsy proven PCa were enrolled in this study. The median age was 68 years (range: 45–85 years), whereas the median PSA level was 7.82 ng/ml. Patients’ characteristics are reported in Table 1. Forty-six out of 85 patients were examined on a PET/MRI scanner (see an example; Figure 2, top panel) and 39/85 on a PET/CT scanner (see an example; Figure 2, bottom panel).

**TABLE 1:** Patients’ characteristics.

Statistics	
No. of patients	85
Median age, years	68 (range: 45–85)
Median PSA, ng/ml	7.82 (range: 1.72–1263)
**ISUP grade, no. (%)**	
1	3 (3.6%)
2	9 (10.6%)
3	17 (20.0%)
4	20 (23.5%)
5	29 (34.1%)
Unknown	7 (8.2%)
**Scanner**	
PET/MRI	46
PET/CT	39

### 3.2. CNN performance

**FIGURE 3:**
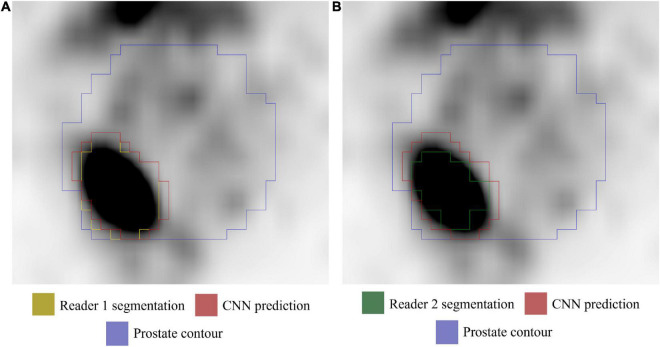
Axial ^68^Ga-PSMA PET image (image windowing SUVmin-max: 0–5). **(A)** GTV-Exp 1 lesion contour (yellow). **(B)** GTV-Exp 2 lesion contour (green). The GTV-CNN contour is shown in red and the prostate contour in purple.

**FIGURE 4:**
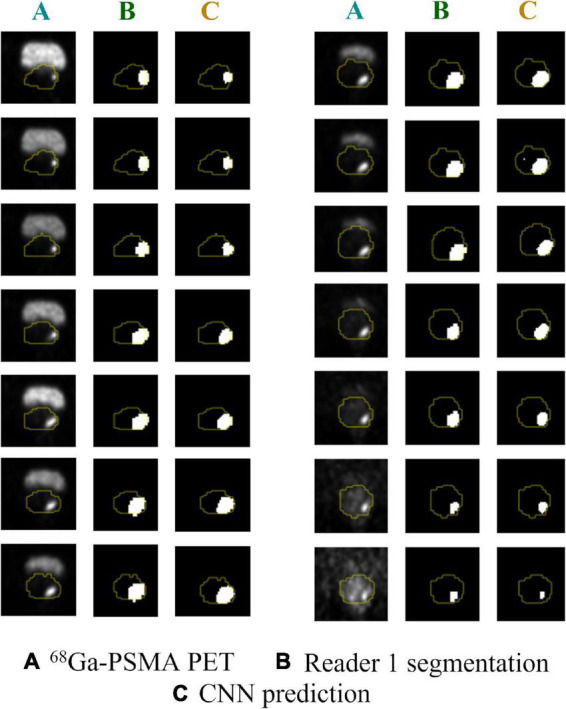
Predicted vs. actual lesion contours in sequential ^68^Ga-PSMA PET slices. **(A)** Original ^68^PSMA PET images; **(B)** ground truth GTV-Exp 1 contours; **(C)** CNN predicted contours. Prostate contours are shown in yellow.

Analyses were performed on PET volumes resampled with tri-linear interpolation and then repeated on images resampled using B-spline interpolation. The results based on tri-linear interpolation are reported here, while Supplementary Table 1 contains the results using B-spline interpolation for voxel resampling. The trained CNN, when validated on the lesion volumes manually contoured by the first reader (GTV-Exp 1), reached a median DSC = 0.74 (range: 0.07–0.93). When the ground truth label was drawn without fixed thresholding of voxel values by the second reader (GTV-Exp 2), the CNN obtained a median DSC = 0.69 (range: 0.07–0.96). However, this difference was not statistically significant (*P* value > 0.05). Using tri-linear or B-spline interpolation did not affect model’s performance (*P* value > 0.05). See Table 2 for a detailed description of CNN model performance, and Figure 3 for a representative image. To better show the performance of the CNN, additional segmentation results for sequential ^68^Ga-PSMA-PET slices are shown in Figure 4. Moreover, no statistically significant differences were identified in the volumes of the intraprostatic tumor lesions defined by the expert Nuclear Medicine physicians and those predicted by the CNN (*P* value > 0.05, Table 3).

**TABLE 2:** External validation of the CNN performance.

	Mean DSC ± SD		Median DSC (range)	
	GTV-Exp 1 vs. GTV-CNN	GTV-Exp 2 vs. GTV-CNN	GTV-Exp 1 vs. GTV-CNN	GTV-Exp 2 vs. GTV-CNN
All	0.70 ± 0.18	0.67 ± 0.20	0.74 (0.07 – 0.93)	0.69 (0.07 – 0.96)
PET/MRI	0.69 ± 0.18	0.64 ± 0.21	0.72 (0.07 – 0.93)	0.68 (0.07 – 0.96)
PET/CT	0.71 ± 0.19	0.70 ± 0.19	0.77 (0.10 – 0.90)	0.75 (0.10 – 0.91)

Mean and median performance of the CNN for the automatic segmentation of intraprostatic cancer lesions considering the contouring made by reader 1 (Exp 1) and reader 2 (Exp 2) as ground truth.

**TABLE 3:** Gross tumor volume.

	GTV-Exp 1	GTV-Exp 2	GTV-CNN
All	12.23 ± 15.7 ml	12.15 ± 16.2 ml	16.55 ± 18.6 ml
PET/MRI	13.75 ± 18.3 ml	12.45 ± 18.3 ml	17.70 ± 20.1 ml
PET/CT	10.45 ± 12.1 ml	11.80 ± 13.5 ml	15.20 ± 17.0 ml

Mean volume, and standard deviation, of the intraprostatic cancer lesion (GTV) defined by Exp 1, Exp 2 and by the CNN.

The DSC obtained by comparing the PCa lesion contouring manually defined by the two expert Nuclear Medicine physicians was 0.73 (range: 0.25–0.92).

No statistically significant differences in CNN performance between PET/MRI and PET/CT images, regardless of the method used to visualize and contour PET images (*P* value > 0.05 for both GTV-Exp 1 and GTV-Exp 2) were observed. Conversely, a positive correlation was found between DSC and GTV-Exp (*r* = 0.43, *P* value < 0.001 and *r* = 0.44, *P* value < 0.001 for GTV-Exp 1 and GTV-Exp 2, respectively), meaning that the CNN produced more accurate segmentations for bigger lesions.

## 4. Discussion

In the present work, an external validation of a CNN for the automatic segmentation of intraprostatic cancer lesions on ^68^Ga-PSMA PET images previously presented by Kostyszyn and colleagues (18) has been performed. In our cohort, the trained CNN model reached a median DSC = 0.74 and its performance was independent from the imaging technique, PET/MRI or PET/CT, used to acquire PET images.

^68^Ga-PSMA PET is widely used for the characterization of PCa in different settings and has been recently included into the EAU-ESTRO-SIOG guidelines for high-risk PCa staging (2). Several studies have been reported showing the potential utility of quantitative features extracted from ^68^Ga-PSMA PET images for the characterization of the disease (9–11). Considering the role of PSMA PET, a possible forthcoming application might be its use in the diagnosis of clinically significant PCa, including biopsy guidance in patients with equivocal mp-MRI findings (6, 24).

Accurate contouring of intraprostatic GTV is required as the starting point both for biopsy guidance and for radiomic analysis. However, this procedure is extremely time consuming and affected by inter-reader heterogeneity, often resulting in non-replicable segmentations (12). Several CNNs have already been proposed for GTV segmentation in other oncological settings (19–21), bearing the potential to become a “one-stop shop” for improving the diagnostics and prognostics of various tumors, including PCa (25).

Kostyszyn and colleagues were the first to generate a CNN for the automatic segmentation of intraprostatic cancer lesions on PSMA PET images (18). This study was a joint effort of 3 different Institutions, 2 in Germany and 1 in China. The generated model was trained on 152 patients, employing images acquired with different tomographs in different centers (1 in Germany and 1 in China). However, only 20 patients in the testing cohort were studied at an external institution (center 3, Germany) not used for training, limiting conclusions regarding the model’s generalizability.

Validation of AI models in external, independent cohorts is crucial to assess their robustness and, consequently, their potential utility. In our study, we tested the model generated by Kostyszyn and colleagues on a cohort of 85 patients examined with ^68^Ga-PSMA PET at our Institution. Considering that image pre-processing can affect the model performance, as previously described in Kostyszyn et al. (18), all pre-processing steps were performed in agreement with the reference work. However, in the present study, images were independently reviewed by two Nuclear Medicine physicians. The first one (Exp 1) followed the instruction given in Kostyszyn et al. (18), while the second (Exp 2) was not informed on how images were viewed in the reference work, thus avoiding the introduction of any bias relative to the adopted segmentation method.

The trained CNN model achieved a moderately good performance on our cohort, reaching at best a median DSC = 0.74. Interestingly, results were independent of the modality used to acquire the images, despite the model being originally trained only on PET/CT images, as well as of the windowing of voxel values used when defining the ground truth labels. These results suggest that using images acquired with several different PET/CT scanners for training contributed to increasing model robustness. Moreover, it has been shown that the thresholding of voxel values SUVmin-max: 0-5 yields relatively stable contouring, as also reported in a previous work of the same group. (12). However, the CNN performance was affected by the volume of the ground truth labels (GTV-Exp 1 and GTV-Exp 2), resulting in more accurate segmentations for bigger lesions.

The main limitation of this study is its monocentric nature, as PET images were acquired in a single Institution. However, as our center was not included in the reference work of Kostyszyn et al., our population represents a large independent and external testing cohort. Moreover, we included patients examined both with PET/CT (*N* = 39) or PET/MRI (*N* = 46), this could have potentially affected the results, but also allowed the comparison of model performance on images acquired with different modalities. Post-hoc analyses showed that no statistically significant differences in CNN performance was observed on images acquired with either PET/MRI or PET/CT. Nineteen patients studied with ^18^F-PSMA were included in the paper presented by Kostyszyn et al. All patients considered in this work underwent ^68^Ga-PSMA PET, therefore, future studies are needed to assess the model’s generalizability to ^18^F-PSMA PET findings.

In conclusion, the trained and publicly available CNN model presented by Kostyszyn et al. (18) yields fairly accurate contouring of intraprostatic cancer lesions on ^68^Ga-PSMA PET images that could be used as a starting point for quantitative analysis using radiomics or deep learning approaches. Nonetheless, more robust performance is needed for the generation of AI-based decision support technologies that can be used and exploited in daily clinical practice.

## Data availability statement

Data supporting the conclusions of this article will be made available by the corresponding author upon reasonable request.

## Ethics statement

The studies involving human participants were reviewed and approved by Ethic Committee of IRCCS San Raffaele Scientific Institute. Written informed consent for participation was not required for this study in accordance with the national legislation and the institutional requirements.

## Author contributions

SG and MP: conceptualization and study design. SG, SM, and CB: formal analysis. PM, AS, FB, LJ-L, EP, TR, and GB: images acquisition and interpretation. SM, SG, and IN: data curation. SG and SM: writing—original draft preparation. PM, PS, FD, and MP: writing—review and editing. PS, FD, and MP: supervision. All authors read and approved the submitted version of the manuscript.

## References

[1] World Cancer, Research Fund International [WCRF]. *Cancer statistics.* (2020). Available online at: https://www.wcrf.org/cancer-trends/prostate-cancer-statistics/ (accessed November 22, 2022).

[2] Mottet N Cornford P van den Bergh R Briers E de Santis M Gillessen S EAU-EANM-ESTRO-ESUR-ISUP-SIOG guidelines on prostate cancer. *Eur Assoc Urol. Proceedings of the EAU annual congress Amsterdam 2022. ISBN 978-94-92671-16-5 (accessed October 24, 2022)*, Amsterdam (2022).

[3] Donato P Morton A Yaxley J Ranasinghe S Teloken P Kyle S ^68^Ga-PSMA PET/CT better characterises localised prostate cancer after MRI and transperineal prostate biopsy: is ^68^Ga-PSMA PET/CT guided biopsy the future? *Eur J Nucl Med Mol Imaging.* (2020) 47:1843–51. 31912257 10.1007/s00259-019-04620-0

[4] Rhee H Thomas P Shepherd B Gustafson S Vela I Russell P Prostate specific membrane antigen positron emission tomography may improve the diagnostic accuracy of multiparametric magnetic resonance imaging in localized prostate cancer. *J Urol.* (2016) 196:1261–7. 27220897 10.1016/j.juro.2016.02.3000

[5] Ferraro D Becker A Kranzbühler B Mebert I Baltensperger A Zeimpekis K Diagnostic performance of ^68^Ga-PSMA-11 PET/MRI-guided biopsy in patients with suspected prostate cancer: a prospective single-center study. *Eur J Nucl Med Mol Imaging.* (2021) 48:3315–24. 10.1007/s00259-021-05261-y 33620559 PMC8426229

[6] Kawada T Yanagisawa T Rajwa P Sari Motlagh R Mostafaei H Quhal F Diagnostic performance of prostate-specific membrane antigen positron emission tomography-targeted biopsy for detection of clinically significant prostate cancer: a systematic review and meta-analysis. *Eur Urol Oncol.* (2022) 5:390–400. 10.1016/j.euo.2022.04.006 35715320

[7] Emmett L Buteau J Papa N Moon D Thompson J Roberts M The additive diagnostic value of prostate-specific membrane antigen positron emission tomography computed tomography to multiparametric magnetic resonance imaging triage in the diagnosis of prostate cancer (PRIMARY): a prospective multicentre study. *Eur Urol.* (2021) 80:682–9. 10.1016/j.eururo.2021.08.002 34465492

[8] Liu C Liu T Zhang Z Zhang N Du P Yang Y ^68^Ga-PSMA PET/CT combined with PET/Ultrasound-guided prostate biopsy can diagnose clinically significant prostate cancer in men with previous negative biopsy results. *J Nucl Med.* (2020) 61:1314–9. 32034111 10.2967/jnumed.119.235333 PMC7456174

[9] Ghezzo S Bezzi C Presotto L Mapelli P Bettinardi V Savi A State of the art of radiomic analysis in the clinical management of prostate cancer: a systematic review. *Crit Rev Oncol Hematol.* (2022) 169:103544. 10.1016/j.critrevonc.2021.103544 34801699

[10] Solari E Gafita A Schachoff S Bogdanoviæ B Villagrán Asiares A Amiel T The added value of PSMA PET/MR radiomics for prostate cancer staging. *Eur J Nucl Med Mol Imaging.* (2022) 49:527–38. 10.1007/s00259-021-05430-z 34255130 PMC8803696

[11] Papp L Spielvogel C Grubmüller B Grahovac M Krajnc D Ecsedi B Supervised machine learning enables non-invasive lesion characterization in primary prostate cancer with [^68^Ga]Ga-PSMA-11 PET/MRI. *Eur J Nucl Med Mol Imaging.* (2021) 48:1795–805. 10.1007/s00259-020-05140-y 33341915 PMC8113201

[12] Zamboglou C Fassbender T Steffan L Schiller F Fechter T Carles M Validation of different PSMA-PET/CT-based contouring techniques for intraprostatic tumor definition using histopathology as standard of reference. *Radiother Oncol.* (2019) 141:208–13. 10.1016/j.radonc.2019.07.002 31431386

[13] SIEMENS. *Auto ID.* (2022). Available online at: https://www.siemens-healthineers.com/molecular-imaging/news/auto-id-for-pet-ct (accessed November 24, 2022).

[14] MIM. *Contour protege AI.* (2022). Available online at: https://www.mimsoftware.com/radiation-oncology/contour-protegeai?utm_source=google_ads&utm_medium=ppc&utm_term=&utm_campaign=MIM+Maestro+Europe&hsa_src=g&hsa_acc=2475176161&hsa_ver=3&hsa_ad=538389917704&hsa_cam=1806236075&hsa_grp=127002588378&hsa_net=adwo (accessed November 24, 2022).

[15] Ronneberger O Fischer P Brox T. U-net: convolutional networks for biomedical image segmentation. In: Navab N Hornegger J Wells W Frangi A editors. *Medical image computing and computer-assisted intervention – MICCAI 2015.* Berlin: Springer (2015). p. 234–41.

[16] Lin G Milan A Shen C Reid I. Refinenet: multi-path refinement networks for high-resolution semantic segmentation. *2017 IEEE conference on computer vision and pattern recognition (CVPR).* Piscataway, NJ: IEEE (2017). p. 5168–77. 10.1109/TPAMI.2019.2893630

[17] Liu C Gardner S Wen N Elshaikh M Siddiqui F Movsas B Automatic segmentation of the prostate on CT images using deep neural networks (DNN). *Int J Radiat Oncol.* (2019) 104:924–32. 10.1016/j.ijrobp.2019.03.017 30890447

[18] Kostyszyn D Fechter T Bartl N Grosu A Gratzke C Sigle A Intraprostatic tumor segmentation on PSMA PET images in patients with primary prostate cancer with a convolutional neural network. *J Nucl Med.* (2021) 62:823–8. 10.2967/jnumed.120.254623 33127624 PMC8729869

[19] Wang J Lu J Qin G Shen L Sun Y Ying H Technical note: a deep learning-based autosegmentation of rectal tumors in MR images. *Med Phys.* (2018) 45:2560–4. 10.1002/mp.12918 29663417

[20] Lin L Dou Q Jin Y Zhou G Tang Y Chen W Deep learning for automated contouring of primary tumor volumes by MRI for nasopharyngeal carcinoma. *Radiology.* (2019) 291:677–86. 10.1148/radiol.2019182012 30912722

[21] Huang B Chen Z Wu P Ye Y Feng S Wong C Fully automated delineation of gross tumor volume for head and neck cancer on PET-CT using deep learning: a dual-center study. *Contrast Media Mol Imaging.* (2018) 2018:8923028. 10.1155/2018/8923028 30473644 PMC6220410

[22] Ramspek C Jager K Dekker F Zoccali C van Diepen M. External validation of prognostic models: what, why, how, when and where? *Clin Kidney J.* (2021) 14:49–58. 10.1093/ckj/sfaa188 33564405 PMC7857818

[23] R Core Team. *R: a language and environment for statistical computing.* Vienna: R Foundation for Statistical Computing (2022).

[24] Margel D Bernstine H Groshar D Ber Y Nezrit O Segal N Diagnostic performance of ^68^Ga Prostate-specific membrane antigen PET/MRI compared with multiparametric MRI for detecting clinically significant prostate cancer. *Radiology.* (2021) 301:379–86. 34463555 10.1148/radiol.2021204093

[25] Shimizu H Nakayama K. Artificial intelligence in oncology. *Cancer Sci.* (2020) 111:1452–60. 32133724 10.1111/cas.14377 PMC7226189

