# Supplementary material for: External validation of a convolutional neural network for the automatic segmentation of intraprostatic tumor lesions on 68Ga-PSMA PET images
Source: Front Med (Lausanne). 2023 Feb 23;10:1133269. doi: 10.3389/fmed.2023.1133269 (PMC9995820; doi:10.3389/fmed.2023.1133269)
Supplement: Supplementary file 1 [file Table_1.DOCX]

Supplementary Material

# Supplementary Tables

Supplementary Table 1. External validation of the CNN performance after B-spline interpolation

|  | **Mean DSC ± SD** | | **Median DSC (range)** | |
| --- | --- | --- | --- | --- |
|  | **GTV-Exp 1 vs GTV-CNN** | **GTV-Exp 2 vs GTV-CNN** | **GTV-Exp 1 vs GTV-CNN** | **GTV-Exp 2 vs GTV-CNN** |
| **All** | 0.69 ± 0.18 | 0.66 ± 0.20 | 0.73 (0.10-0.93) | 0.69 (0.10-0.96) |
| **PET/MRI** | 0.68 ± 0.18 | 0.64 ± 0.21 | 0.71 (0.13-0.93) | 0.66 (0.12-0.96) |
| **PET/CT** | 0.71 ± 0.18 | 0.70 ± 0.19 | 0.76 (0.10-0.90) | 0.74 (0.10-0.90) |
| Mean and median performance of the CNN for the automatic segmentation of intraprostatic cancer lesions considering the contouring made by reader 1 (Exp 1) and reader 2 (Exp 2) as ground truth | | | | |

Supplementary Table 2. Gross tumor volume after B-spline interpolation

|  | **GTV-Exp 1** | **GTV-Exp 2** | **GTV-CNN** |
| --- | --- | --- | --- |
| **All** | 12.23 mL ± 15.7 mL | 12.15 mL ± 16.2 mL | 16.58 mL ± 18.3 mL |
| **PET/MRI** | 13.75 mL ± 18.3 mL | 12.45 mL ± 18.3 mL | 17.71 mL ± 19.7 mL |
| **PET/CT** | 10.45 mL ± 12.1 mL | 11.80 mL ± 13.5 mL | 15.24 mL ± 16.7 mL |
| Mean volume, and standard deviation, of the intraprostatic cancer lesion (GTV) defined by Exp 1, Exp 2 and by the CNN | | | |
